# Postoperative complications do not influence the pattern of early lung function recovery after lung resection for lung cancer in patients at risk

**DOI:** 10.1186/1749-8090-9-92

**Published:** 2014-05-19

**Authors:** Maja Ercegovac, Dragan Subotic, Vladimir Zugic, Radoslav Jakovic, Dejan Moskovljevic, Slavisa Bascarevic, Natasa Mujovic

**Affiliations:** 1 Clinic for thoracic surgery, Clinical center of Serbia, University of Belgrade school of medicine, Visegradska 26, 11000 Belgrade, Serbia; 2 Clinic for pulmonology, Clinical center of Serbia, University of Belgrade school of medicine, Belgrade, Serbia; 3 Service of physical therapy and rehabilitation, Clinical center of Serbia, University of Belgrade school of medicine, Belgrade, Serbia

**Keywords:** Lung cancer, Resection, Complications, Lung function

## Abstract

**Background:**

The pattern and factors influencing the lung function recovery in the first postoperative days are still not fully elucidated, especially in patients at increased risk.

**Methods:**

Prospective study on 60 patients at increased risk, who underwent a lung resection for primary lung cancer. Inclusion criteria: complete resection and one or more known risk factors in form of COPD, cardiovascular disorders, advanced age or other comorbidities. Previous myocardial infarction, myocardial revascularization or stenting, cardiac rhythm disorders, arterial hypertension and myocardiopathy determined the increased cardiac risk. The severity of COPD was graded according to GOLD criteria. The trend of the postoperative lung function recovery was assessed by performing spirometry with a portable spirometer.

**Results:**

Cardiac comorbidity existed in 55%, mild and moderate COPD in 20% and 35% of patients respectively. Measured values of FVC% and FEV_1_% on postoperative days one, three and seven, showed continuous improvement, with significant difference between the days of measurement, especially between days three and seven. There was no difference in the trend of the lung function recovery between patients with and without postoperative complications. Whilst pO_2_ was decreasing during the first three days in a roughly parallel fashion in patients with respiratory, surgical complications and in patients without complications, a slight hypercapnia registered on the first postoperative day was gradually abolished in all groups except in patients with cardiac complications.

**Conclusion:**

Extent of the lung resection and postoperative complications do not significantly influence the trend of the lung function recovery after lung resection for lung cancer.

## Background

Patient’s fitness for the planned extent of lung resection for non small cell lung cancer (NSCLC) represents one of the main elements of the preoperative selection [1].

Currently, the preoperative lung function assessment is focused to the prediction of postoperative ventilatory function and to the estimate of cardiorespiratory reserve. Numerous studies evaluated late effects of lung resection (3–6 months after surgery), related to quality of life, length of survival and exercise tolerance. It is now established that predicted postoperative FEV_1_ (ppoFEV_1_) is accurate in predicting FEV_1_ 3–6 months after surgery, but in the same time it is likely to overestimate the FEV_1_ in the initial post-operative days, when, in fact, most complications occur. It was recently demonstrated that, on the first post-operative day after lobectomy, for example, the measured FEV_1_ may be 30% lower than predicted [2]. Furthermore, in most of the studies of this type, patients without major comorbidity are analysed together with high risk patients.

That is the reason why the aim of this study was to analyze trends of the lung function changes in patients at risk in the immediate postoperative course, regardless of the presence and type of postoperative complications.

## Methods

Prospective study on 60 patients at increased risk, who underwent a lung resection for primary non-small cell lung cancer in a recent one year period.

Inclusion criteria: complete resection and one or more known risk factors in the form of COPD, cardiovascular disorders, advanced age, or other comorbidities.

### Preoperative assessment

Preoperative lung cancer staging was done in a usual manner.

Cardiological evaluation consisted of physical examination, ECG, echocardiography and/or coronarography according to need as suggested by current recommendations [3]. Previous myocardial infarction, myocardial revascularization or stenting, cardiac rhythm disorders, arterial hypertension and myocardiopathy determined the increased cardiac risk.

Other associated comorbidities were registered as diabetes, thyroid or other endocrinological disorders, peripheral vascular disease, chronic renal insufficiency, previous cerebrovascular infarctions and nutritional status. The latter was expressed as body mass index (BMI) with cut off value of <18 and >30 for malnutrition and excessive obesity respectively.

Preoperative lung function testing consisted of standard spirometry (FEV_1_, VC, Tiffeneau index, FEF_50_, FEF_25_), measurement of diffusion with determination of transfer factor (DL_CO_) and diffusion coefficient.

In COPD patients, respiratory function tests were performed before and after combined bronchodilator therapy. The best measured values were taken as baseline for the calculation of postoperative FEV_1_. The severity of COPD was graded according to GOLD criteria [4] The existence and extent of bullous emphysema was assessed based on measurement of the lung tissue density on high resolution CT.

In patients with moderate COPD and/or DL_CO_ <70% and anticipated pneumonectomy, perfusion lung scintigraphy was done in order to calculate the predicted postoperative FEV_1_ (_ppo_FEV_1_)_._ In these patients, ppo FEV1% was calculated on the basis of preoperative FEV_1_% and percent perfusion of remaining lung [5]. If the high resolution CT revealed bullous emphysema either in the tumour bearing or other lobes, perfusion lung scintigraphy was done as well, even before lobectomy. In that case, calculation of the ppoFEV_1_ was done by using a stepwise method [6]:

$$\begin{array}{l} \mathrm{ppoLoss}\;\mathrm{in}\;{\mathrm{FEV}}_{1}=\mathrm{preop}.{\mathrm{FEV}}_{1}x\%\mathrm{perfusion}\;\mathrm{of}\;\mathrm{the}\\ \;\mathrm{Tu}\;\mathrm{bearing}\;\mathrm{lung}\;x\;n/n_{1} \end{array}$$

$${\mathrm{ppoFEV}}_{1}={\mathrm{preopFEV}}_{1}–\mathrm{ppoLoss}\;\mathrm{in}\;{\mathrm{FEV}}_{1}$$

n: number of segments to be resected; n_1_: total number of segments of the diseased lung

In all candidates for lobectomy, calculation of ppoFEV_1_ was done by using Nakahara formula [7] that includes the number of obstructed lung segments, with additional calculation by using a perfusion lung scintigraphy, if associated COPD existed, as described above.

The COPD index was calculated as sum of decimal values of FEV_1_% and FEV_1_%/FVC% [8].

In patients at increased cardiac risk, either isolated, or associated with COPD, oxygen consumption was determined on ergometer bicycle (system VIASYS Master Screen CPX) according to institutional protocol. During this test, continuous registration of lung ventilation, heart rate, and arterial tension at rest and during the effort was done. The incremental load increase consisted of 20 W increase each 3 minutes until the submaximal load was acheved as manifested by submaximal heart rate, switching to the anaerobic metabolism (RER >1) or critical decrease of respiratory reserve. The test was aborted in case of severe dyspnea, retrosternal pain, vertiginous symptoms, severe claudications or abrupt arterial pressure rise. A cut off value of 15 ml/kg/min was accepted as a lower limits for safe pneumonectomy and lobectomy., with values <10 ml/kg/min being prohibitive for any anatomical resection.

### Operative procedure

In all patients lung resections were performed through open approach - muscle sparing thoracotomy with extension to full posterolateral thoracotomy according to the need. Incomplete or absent fissures were divided with stappling devices. Bronchial staplers were used for lobectomy, whilst manual bronchial closure was the standard technique for pneumonectomy. Systematic lymphadenectomy was done in all patients. Two chest tubes under active suction (20 cmH_2_O) were routinely used with successive removal between the 3th and 7th postoperative days.

All patients underwent postoperative respiratory rehabilitation according to institutional protocol (inhalation with secretolitics and/or bronchidlators, forced breathing, early mobilization). In case of imminent athelectasis, with radiographic aspect of mediastinal displacement towards the operated side, intensified physiotherapy was the first step, followed by bedside fiberbronchoscopic aspiration after 24 hours at maximum, in case of treatment failure or progression of athelectasis.

In addition to the usual policy of two chest tubes aspiration after lobectomy, in some patients with well developed fissures, only one chest tube is used. After pneumonectomy, 24 h underwater-seal chest tube drainage is a standard institutional practice.

The existence of postoperative air leak is confirmed by the presence of air-bubbles within a drainage bottle with the chest tube under active aspiration. In such patients, the degree of the lung expansion is checked daily or in 2-days intervals by performing in-bed radiographies under active suction. In patients with a prolonged air leak, the operative chest tubes are always removed after the 7th postoperative day at maximum, and in case of persisting lung collapse, a new, narrower caliber chest tube (Fr 18–20 max. diameter) is reinserted. The point of the tube reinsertion is selected based on the fluoroscopic aspect.

### Postoperative lung function assessment

The trend of the postoperative lung function recovery was assessed by performing spirometry with a portable spirometer “Viasys Spiro Pro”, with a patient in the sitting position in bed, with chest tubes connected and under maximal analgesia (NSAIDS and tramadol-chloride). Analgetics were administrated in regular intervals during the first 48-72 h, followed by administration upon patient’s request.

Measurement of FVC%, FEV_1_%, VC and PEF _75_ was done on postoperative days one, three and seven.

### Data analysis

The following variables were analysed: age, sex, preoperative and postoperative values of FVC%, FEV_1_%, FEV_1_%/FVC%, FEF_50_, FEF_25_, pCO_2_, pO_2_, DL_CO_, VO_2_ max, COPD index, predicted postoperative FEV_1_%, postoperative complications.

In the current study, operative morbidity and mortality referred to complications or death inside the first 30 postoperative days. Postoperative complications were classified as respiratory (pneumonia, athelectasis, pulmonary embolism, respiratory insufficiency and need for prolonged mechanical ventilation, ARDS/ALI), surgical (prolonged air-leak, postoperative bleeding, pleural effusion, empyema, bronchopleural fistula, operative wound problems) and cardiac (atrial fibrillation, myocardial infarction, congestive heart failure).

Criteria for diagnosis of pneumonia were fever, leukocyte count < 3000 or > 10000, new infiltrates of chest x-ray and/or positive sputum cultures.

Prolonged air-leak was defined as air-leak longer than 7 postoperative days, regardless of the existence of full lung expansion under active suction [1].

The described methodology was approved by the Ethical committee of the University of Belgrade school of medicine.

### Statistics

Statistical analysis consisted of nonparametric and parametric tests (Mann- Whitney U test, Kruskal-Wallis test, Sudent’s t-test, Chi-square test, Pearson coefficient of correlation, ANOVA).

## Results and discussion

### Results

The structure of the analysed group is presented on Table 1. The majority of patients - 42(70%) were male, with 39(65%) of them being active smokers at the time of surgery. The mean/range age for male and female patients was 60.9 ± 8.4/41-77 and 56.9 ± 6.5/44-68 years respectively.

**Table 1:** Patient characteristics

	**n**	**%**
**Sex** (M/F)	42/18	70/30
**Smoking habits** (ns, fs, cs)	8/13/39	13.3/21.7/65
**BMI** (<18.5/18,5-30/>30)	2/49/9	3.3/81.7/15
**Comorbidity** (cardiac/ noncardiac/both)	33/26/21	55/43.3/35
**COPD stage** (0/1/2/3)	27/12/21/0	45/20/35/0
**Disease stage** (I/II/IIIA/IIIB)	22/28/8/2	36.7/46.7/13.3/3.3
**Extent of resection** sublobar/ lobectomy/pneumonectomy.	5/41/14	8.3/68.4/23.3

ns: non smoker; fs: former smoker; cs: current smoker.

Cardiac comorbidities existed in 33(55%), other comorbidities (mainly renal, diabetes mellitus, peripheral vascular) in 26(43.3%) and both cardiac and other comorbidities in 21(35%) patients. Body mass index >30 existed in 9(15%) patients. Mild and moderate COPD according to GOLD criteria, existed in 20% and 35% of patients respectively.

Based on operative patohistology, almost 80% of patients were in stages I and II. Most of the patients underwent a lobectomy, pneumonectomy was done in 14(23.3%), sublobar resection in 5(8.3%) patients.

### Preoperative lung function and postoperative complications

Postoperative complications in the analysed group are presented on Table 2. Surgical complications were more frequent than respiratory and cardiac. All registered respiratory and cardiological complications occurred within the first 7 postopertive days. Air leak, if present at the moment of the lung function measurement, persisted after more than 7 postoperative days as well.

**Table 2:** Postoperative complications

**Surgical complications**	**n**	**%**
No complications	35	58.3
Air-leak	10	**16.7**
Pl.effusion, hemothorax, wound problems, B.pl. fistula	15	25
**Respiratory complications**	n	%
No complications	48	80
Pneumonia	8	**13.4**
Atelectasis	4	**6.6**
**Cardiac complications**	n	%
No complications	50	83.4
Atrial fibrillation	9	**15**
Pulmonary embolism	1	1.6

Among surgical complications, only postoperative haemothorax occurred within the first 3 postoperative days; and did not require rethoracotomy.

Comparison of patients with surgical, respiratory and cardiac postoperative complications with patients without complications, regarding ventilatory function, diffusion, oxygenation and oxygen consumption, did not confirm significant differences (Table 3). Female, compared with male patients (not presented on table), had significantly higher preoperative FVC% (98.9 ± 16.28 vs 112.1 ± 23.69), FEV1% (83.6 ± 18.08 vs 95.9 ± 27.48), COPD index (1.45 ± 0.24 vs. 1.66 ± 0.37) and ppo FEV1% (60.3 ± 15.8 vs 72.2 ± 20.37).

**Table 3:** Preoperative lung function parameters in patients without and with postoperative complications

	**With complications**						**Without complications (4)**		**P**
	***Surgical (1)***		***Respiratory (2)***		***Cardiac (3)***				
	**Mean**	**SD**	**Mean**	**SD**	**Mean**	**SD**	**Mean**	**SD**	
**FVC%**	102.6	21.36	93.8	18.65	102.8	20.23	104.0	18.88	*1,2,3 vs. 4* **> 0.05**
**FEV1%**	86.6	23.05	81.8	24.40	86.8	21.47	87.3	20.31	*1,2,3 vs. 4* **> 0.05**
**FEV1%/FVC**	68.4	11.23	69.1	11.31	67.7	7.99	69.0	10.99	*1,2,3 vs. 4* **> 0.05**
**COPD index**	1.5	0.29	1.5	0.3	1.5	0.25	1.5	0.31	*1,2,3 vs. 4* **> 0.05**
**MEF 50**	52.2	31.4	51.1	36.05	47.3	32.71	49.1	21.77	*1,2,3 vs. 4* **> 0.05**
**MEF 25**	43.6	30.24	41.5	33.01	38.8	26.82	40.9	18.74	*1,2,3 vs. 4* **> 0.05**
**ppo FEV1**	64.2	20.76	63.2	19.79	60.6	21.40	63.0	13.94	*1,2,3 vs. 4* **> 0.05**
**pCO**_**2**_	5.2	0.59	5.3	0.60	5.0	0.67	5.3	0.55	*1,2,3 vs. 4* **> 0.05**
**pO**_**2**_	10.8	1.72	10.2	1.2	9.9	0.91	10.4	0.97	*1,2,3 vs. 4* **> 0.05**
**DLCO**	67.1	14.5	71.3	17.37	71.7	20.10	72.5	18.37	*1,2,3 vs. 4* **> 0.05**
**VO**_**2 **_**max**	18.0	6.52	16.0	5.28	17.4	3.54	17.2	4.82	*1,2,3 vs. 4* **> 0.05**

**FVC%:** forced vital capacity expressed in percent; **FEV1%:** forced expiratory volume in the 1 second expressed in percent; **FEV1%/FVC:** as just explained; **COPD index:** index of chronic obstructive pulmonary disease; **MEF 50:** mean expiratory flow at 50% vital capacity; **MEF 25:** mean expiratory flow at 25% vital capacity; **ppo FEV1:** predicted postoperative FEV_1_; **pCO**_**2:**_ partial pressure of CO_2_ in the arterial blood; **pO**_**2:**_ partial pressure of O_2_ in the arterial blood; **DLCO:** diffusing capacity for carbon monoxide; **VO**_**2 **_**max:** maximal oxygen consumption.

### Postoperative changes of ventilatory function and oxygenation

As presented on Table 4, a clear trend of improvement of both FVC% and FEV_1_% was registered in patients with surgical and respiratory complications, whilst in patients with cardiac complications, the same trend existed only the FVC(%). In these patients, beside evident gradual improvement of FEV_1_, the borderline improvement was registered only between days one and three. The same trend existed in patients without postoperative complications.

**Table 4:** Lung function parameters on days 1, 3, 7 in pts with surgical, respiratory and cardiac complications and without postoperative complications

***Complications***	**Analysed parameter**	**Postoperative day**						**P value**
		**1**		**3**		**7**		
		**Mean**	**SD**	**Mean**	**SD**	**Mean**	**SD**	
*Surgical*	FVC(%)	48.0	17.46	57.2	17.6	69.5	21.87	d1:3: p < 0.001; d3:7: p < 0.001
	FEV_1_(%)	44.0	15.14	52.2	17.59	62.0	21.10	d1:3: p < 0.001; d3:7: p < 0.001
	pO_2_	16.8	5.72	13.5	4.81	12.5	3.52	d1:2 p < 0.05; d2:3 p > 0.05
	pCO_2_	5.6	0.86	5.6	0.68	5.4	0.76	d1:2 p = 0.05; d2:3 p > 0.05
*Respiratory*	FVC(%)	3.87	10.19	46.5	11.0	62.1	15.26	d1:3: p < 0.001; d3:7: p < 0.001
	FEV_1_(%)	38.6	11.47	44.3	13.3	59.5	18.52	d1:3: p < 0.05; d3:7: p < 0.001
	pO_2_	13.8	3.73	13.4	4.62	11.5	3.75	d1:2 p > 0.05; d2:3 p > 0.05
	pCO_2_	5.8	1.08	5.5	0.75	5.4	0.55	d1:2 p > 0.05; d2:3 p > 0.05
*Cardiac*	FVC(%)	46.1	17.31	52.7	19.33	62.9	19.18	d1:3: p < 0.05; d3:7: p < 0.05
	FEV_1_(%)	43.7	13.85	49.9	17.27	58.9	15.65	d1:3: p = 0.05; d3:7: p > 0.05
	pO_2_	17.5	6.18	18.6	8.38	13.2	4.06	d1:2 p > 0.05; d2:3 p < 0.05
	pCO_2_	5.3	0.86	5.5	0.95	5.6	0.93	d1:2 p > 0.05; d2:3 p > 0.05
*No complications*	FVC(%)	53.3	16.69	60.4	18.27	68.7	16.84	d1:3: p < 0.05; d3:7: p < 0.05
	FEV_1_(%)	49.4	12.43	53.5	13.20	61.1	13.98	d1:3: p < 0.05; d3:7: p < 0.001
	pO_2_	17.1	5.28	14.7	4.03	13.2	3.71	d1:2 p < 0.05; d2:3 p > 0.05
	pCO_2_	5.7	0.75	5.6	0.49	5.4	0.65	d1:2 p > 0.05; d2:3 p = 0.05

**Figure 1:**
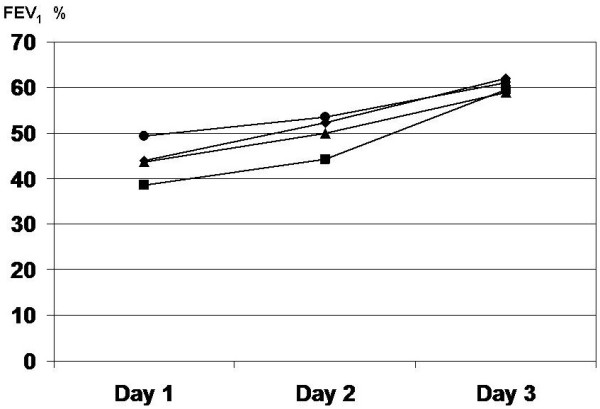
**The trend of postoperative FEV**_**1 **_**change in patients with and without complications.** ♦ surgical complications, ■ cardiac complications, ▲respiratory complications, ● no complications.

The trend of the postoperative FEV_1_ change is presened on the Figure 1. As can be seen, in patients with respiratory complications, the improvement between days three and 7 was greater than in patients of other types of complications and without complications.

The aforementioned trend existed in patients with lobectomy and pneumonectomy, with no differences depending of the site of lobectomy (not shown on graph).

As mentioned before, baseline values for FVC% and FEV1% were higher in women. Postoperative changes of these parameters were almost identical in males and females (not shown on graph).

**Figure 2:**
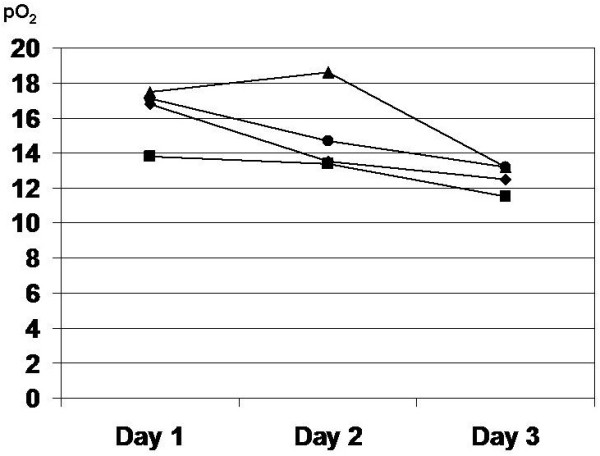
**The trend of postoperative changes of pO**_**2**_**.** ♦ surgical complications, ■ cardiac complications, ▲respiratory complications, ● no complications.

**Figure 3:**
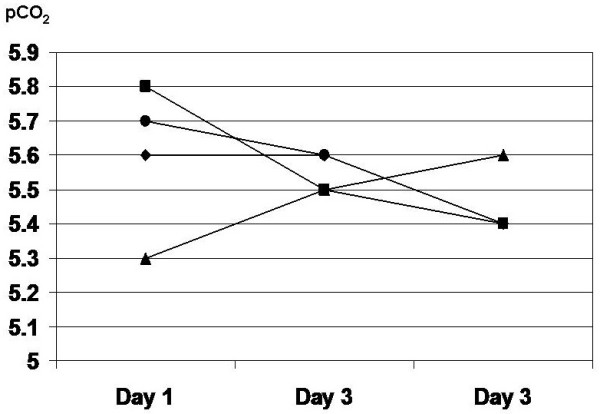
**The trend of postoperative changes of pCO**_**2**_**.** ♦ surgical complications, ■ cardiac complications, ▲respiratory complications, ● no complications.

Concerning oxygenation in the arterial blood, pO_2_ was decreasing during the first three postoperative days in a roughly parallel fashion in patients with respiratory, surgical complications and in patients without complications. In patients with cardiac complications, a steep drop occurred at the moment of the complication onset (Figure 2). A slight hypercapnia registered on the first postoperative day was gradually abolished in all groups except in patients with cardiac complications (Figure 3).

In an attempt to avoid a potential selection bias, we calculated Thoracoscore both for the analysed group of patients at high risk and for the well matched control group of operated patients with a low risk, as assessed according to the same criteria, and compared these groups. A significant difference was found between the groups in relation to Thoracoscore (Table 5).

**Table 5:** Thoracoscore in the high and low-risk groups

**Thoracoscore**							
	***Mean***	***SD***	***95% CI***		***Mann Whitney- U***	***Wilcoxon W***	***Asymp.Sig. (2-tailed)***
			***Lower***	***upper***			
High risk	2.58	2.59	1.91	3.25			
Low risk	1.02	0.51	0.89	1.16	1077.5	2847.5	0.000

### Discussion

One of the main findings of the current study is a significant increase in postoperative FEV1 and FVC values between days 1. and 3. and between days 3 and 7. irrespectively of complications. Such a finding needs to be discussed from different aspects.

First, such a finding may seem unexpected, having in mind that the air leak, occurring in up to 21.7% pts after lobectomy, irrespectively of the performed technique, usually reaches its maximum just during the first 3–4 postoperative days [9]. The possible explanation for the obtained trend of the postoperative ventilatory function improvement is the fact that, in the analysed group, full lung expansion existed despite the air leak in all patients, as assessed on the day of operation and on the first postoperative day by chest radiographies with chest tubes connected. It can only be speculated whether the suction, applied to chest tubes, representing our institutional policy, through the decrease of differential pleural pressure, possibly led to decreased work of breathing, at least after upper lobectomies, as suggested in some recent studies [10]. In the analysed group, the minimally negative pressure (ranging 10–40 cm H_2_O) enabling full apposition of both pleural surfaces, was applied to the chest tubes.

Second, it was also demonstrated that the site of lobectomy may influence the postoperative pulmonary function, with the ventilatory function being better preserved and oxygen consumption being better than predicted after lower/middle than after upper lobectomy [11]. In the present study, the aforementioned trend existed in the entire group, consisting of both COPD and non COPD patients and without differences depending of the site of lobectomy. Results of several reports showing that the lung function can be better preserved after upper lobectomy in COPD patients [12], can be counterweighted by recent findings that the observed postoperative loss in FEV_1_ may exceed the predicted loss after upper lobectomies in COPD patients [13]. It means that COPD strongly influences FEV_1_ at both the early and late terms after upper lobectomy, so that the exact way of it’s influence to the early postoperative lung function preservation still has not been fully elucidated. Based on estimated CT scan tissue density, bullous emphysema was present in 4 pts. Therefore lung volume reduction effect, possibly existing in less than 10% of patients in analysed group, can not be considered as significant contributor to observed trends in postoperative changes of ventilatory function parameters.

Third, our finding that the trend of the early ventilatory function improvement existed also after pneumonectomy, supports recent suggestions that, in patients undergoing pneumonectomy, the ppoFEV1 can underestimate the actual poFEV1 by an average of 500 ml [14]. The smaller loss in FEV1 and greater decrease of hyperinflation after pneumonectomy in COPD vs non-COPD patients means that prediction of the postoperative lung function in candidates for pneumonectomy with limited lung function, can be done in a similar way than in COPD patients undergoing a lobectomy: COPD patients are likely to do a little bit better postoperatively than predicted [15]. Furthermore, there is widespread belief that sacrifying of the phrenic nerve during pneumonectomy has no major impact to the postoperative lung function. However, it was convincingly demonstrated that, in patients with preserved phrenic nerve and normal diaphragm motion, the postoperative FEV_1_ was significantly better than in patients with either immobile diaphragm or with paradoxical diaphragm motion [16]. In the analysed group, phrenic nerve function has been preserved in all patients undergoing pneumonectomy.

Furthermore, in the analysed group, it seems that mediastinal displacement towards the operated side after pneumonectomy did not influence the described trend, although it could have some impact to the overall ventilatory performance - as it was shown that, in COPD patients it has lung volume reduction effect, whilst in non COPD patients it can cause vicariate emphysema [17]. In the analysed group, major mediastinal displacement was registered in the first 3–7 postoperative days in 4 out of 16 patients after pneumonectomy.

Finally, one of the factors that could contribute to the gradually improving trend of the postoperative ventilatory function, could be the function of the small airways. In most studies that confirmed better postoperative lung function preservation in COPD vs. non-COPD patients, the preoperative function and response of the small airways to medical treatment was not addressed. We also recently confirmed the smaller postoperative loss in FEV_1_ in COPD vs. non-COPD patients, but only after significant improvement of the function of the small airways after combined bronchodilator treatment - the trend that remained unchanged postoperatively [18].

Our finding that the type of postoperative complications did not alter the same trend of the lung function improvement during the first 7 postoperative days, could be of practical benefit and needs particular discussion.

As for the influence of respiratory complications, such a finding can be explained by the fact that postoperative athelectasis, that could certainly lead to the impairment of ventilatory function, existed in only 4 (6.6%) patients and was efficiently solved by bronchoscopic aspiration. Air leak, another factor that could lead to inefficient ventilation, as already mentioned, even when present, was always associated with expanded lung, meaning that the pleural pressure remained negative throughout that period.

Concerning the maintenance of the tend of the ventilatory function improvement and cardiac complications, only the newly discovered atrial fibrillation was included among cardiac complications, being significantly associated with preexisting cardiac comorbidities (atrial fibrillation developed in 8 patients, in only one without previously known cardiac disease). Higher incidence of cardiac comorbidities in our group than in some other reported series, can be partly explained by the fact that hypertension, present in 22% of pts, was considered as cardiac comorbidity [19,20]. Significant number of current (65%) and former smokers (21.7%) also may contribute to high incidence of cardiac diseases in our patients. Cardiac complications could influence the postoperative course by defining the protocol of physiotherapy, switching it towards less intensive, inhalatory treatment. Low number of patienst with atrial fibrillation in our group supports the need for further controlled studies of postoperative cardiac adverse events and lung function recovery on larger patient samples.

There are no studies specifically addressing adjustment of VO_2_ cut-off values according to comorbidity. Currently, basic cut-off values for *V*O2 are >75% predicted (>20 mL · kg ˉ^1^ · minˉ^1^) for pneumonectomy whilst values <35% pred (<10 mL/kg/min) represent a high risk for any resection [21]. Evidence is still not sufficient to recommend cut-off values for lobectomy. The obtained result suggests that even lower than recommended cut-off value of VO_2_ can be accepted for pneumonectomy, in the absence of major cardiac comorbidity. In the present study, VO_2_ ≥ 15 mL/kg/min was accepted as a limit for increased risk for postoperative complications, irrespectively of the extent of resection. Although the VO_2_ reflects the risk of the overall cardiorespiratory risk, its prediction is usually done in presence of cardiac comorbidity and especially in candidates for pneumonectomy.

Measurement of VO_2_ itself, only in presence of cardiac comorbidity can also be put into question, because a recent study on 1067 patients in a 4-year period, did not confirm cardiac comorbidity as a risk factor for operative morbidity and mortality [22]. Our result that in the analysed group, a slightly higher number of patients with VO_2_ < 15 ml/kg/min and respiratory complications existed, supports our policy to determine maximal oxygen consumption also in patients with limited respiratory function. In the analysed group, all patients underwent measurement of the VO_2._

In the analysed group there was no significant difference in preoperative lung function parameters and oxygenation between patients with and without complications.

Although this analysis included only patients with increased risk, such a finding may seem quite unexpected. Given the well established role of preoperative lung function and oxygenation as risk factors for operative morbidity [23,24], our results suggest that their influence may not be dominant in high risk patients. In fact, it is difficult to compare these data with similar studies, because most of the literature data refer to differences in complication rates between patients with vs. patients without respiratory or cardiac comorbidity, not between subgroups of patients at increased risk.

### Study limitations

The analysed group of patients at risk is a quite heterogeneous group, consisting of, different risk types, like, for example, fit patients with previous history of CABG and an elderly patients with COPD, left ventricle systolic function and stroke disease. The obtained significant difference between patients at risk and control group in relation to Thoracoscore, minimizes a potential selection bias, in the same time favoring a more widespread use of this score in practice.

## Conclusion

In brief, based on the obtained results, it can be concluded that the extent of the lung resection and postoperative complications do not necessarily influence the trend of the lung function recovery after lung resection for lung cancer.

## Consent

“Written informed consent was obtained from the patient for the publication of this report and any accompanying images”.

## Abbreviations

FEV1: Forced expiratory volume in the 1st second; ppoFEV1: Predicted postoperative FEV_1_; COPD: Chronic obstructive pulmonary disease; ECG: Electrocardiogram; VC: Vital capacity; FEF50: Forced expiratory flow at 50% VC; FEF25: Forced expiratory flow at 25% VC; GOLD: Global Initiative for Chronic Obstructive Lung Disease; pCO2: Partial pressure of CO_2_ in the arterial blood; pO2: Partial pressure of O_2_ in the arterial blood; DLCO: Diffusion capacity; VO2 max: Maximal oxygen consumption during exercise; CT: Computed tomography; ARDS: Acute respiratory distress syndrome; ALI: Acute lung injury; NSAIDS: Non-steroidal anti inflammatory drugs.

## Competing interest

The authors declare that they have no competing interests.

## Authors’ contributions

ME: study design, surgery, data collection, analysis, literature survey, discussion. DS: study design, surgery, literature survey, discussion. VZ: respiratory function tests, effort studies, data desciption and analysis. RJ: study design, surgery, discussion. DM: surgery, data collection, entering data into the database. SB: surgery, data collection, entering data into the database. NM: preoperative and postoperative physiotherapy, study design and discussion. All authors read and approved the final manuscript.
